# Editorial: Cross-cultural occupational health psychology challenges for the 21st century

**DOI:** 10.3389/fpsyg.2022.970631

**Published:** 2022-08-03

**Authors:** Muddassar Sarfraz, Larisa Ivascu, Syed Ghulam Meran Shah, Awais Farid

**Affiliations:** ^1^School of Management, Zhejiang Shuren University, Hangzhou, China; ^2^Faculty of Management in Production and Transportation, Politehnica University of Timisoara, Timişoara, Romania; ^3^Department of Business Administration, School of Social Sciences, University of Castilla-La Mancha, Ciudad Real, Spain; ^4^Division of Environment and Sustainability, Hong Kong University of Science and Technology, Kowloon, Hong Kong SAR, China

**Keywords:** occupational health and safety, cultural values, psychological challenges, employees' wellbeing, health challenges

## Introduction

In recent years, the science of positive occupational psychology has drastically enhanced the understanding of the elements that have proven beneficial for improving individuals' wellbeing. This burgeoning research field has inevitably deepened the knowledge of organizational psychological prosperity. Occupational health psychology is raising disciplines that profoundly alter the working conditions, thus influencing individuals' wellbeing. However, despite the increasing significance of OP, individuals' have encountered massive health challenges, substantially leading this subject to gain acceleration in different domains.

Thus, due to the expanding significance of this Research Topic, a special issue on the proposed topic: *Cross-Cultural Occupational Health Psychology Challenges for the 21st Century*, has previously been submitted. Under this title, new insights and thoughts have gathered where numerous pioneer studies had heeded the call, significantly supporting the intended proposal with valuable additions. Several papers had been found enriching the literature, thus opening the avenues for the topic development. The Research Topic addresses the questions that have previously remained unclear. This special issue profoundly revisits the cross-cultural boundaries through numerous impressive submissions. This Research Topic includes relevant articles from different grounds (e.g., China, United States, Pakistan, and Macau). It spawned the magnitude of novel approaches, studies, theories, and research in multiple fields such as management sciences, education, health, and construction. Significantly, it presents 11 inspiring ideas increasing the credibility and impact of OHP on individuals' wellbeing. Altogether, it provides a detailed roadmap for professionals by setting a positive course in psychological perception. Notably, the diverse geographical contributions included in the editorial paper extend the scope of the research by providing a brief overview of the following articles.

However, given the explanation, today, the extended prominence of workplace psychological issues has made the individuals experience work-related instability (e.g., emotional exhaustion) (Reh et al., 2021) and turnovers (Yuniasanti et al., 2019). Significantly, these far-reaching consequences on the employees' mental health highlight the role of job satisfaction in combating the progressing health vulnerabilities. Therefore, in explaining this notion, the study featured by Xue et al. reveals that job satisfaction controls the workplace mental manifestation and burnout. Similarly, supporting this phenomenon, Dong and Xu also confirm that workplace emotional complexity drastically influences the employees' psychological construct. It explains that emotional experiences deplete individuals' psychological systems, thus demanding the organizations to control the disrupting episode of extreme human emotions. In particular, the overextending depletion of psychological wellbeing negatively influences the workers' wellbeing (i.e., stress. and anxiety) (Jeon et al., 2018). This drastic decline in mental health decreases the employees' productivity and performance effectiveness. Accordingly, the study proposed by Akutsu et al. states that undisputed importance to employees' mental health poses a magnificent threat to employees' cognition. It illustrates that the competitive working environment (e.g., workaholic) deteriorates the employees' cognition process, thus adversely influencing their health. Altogether, the research article realizes the worth of maintaining employees' welfare, thereby recording positive cognitive outcomes.

Unsurprisingly, positive employee health has become the focal point of most researchers (Fu and Abbas, 2021). Therefore, achieving sustainable development goals influencing individuals' wellbeing has become essential for ensuring individual health. In this regard, Aman et al. state that mental wellbeing in the multi-sector infrastructure projects (i.e., CPEC) needs attention. In recent years, the welfare projects under the China-Pakistan Economic Corridor have considerably elevated the cultural, social, economic, and environmental factors to threaten the residents' lives. However, this extended concern has made this study adopt an optimistic approach to improving the residents' wellbeing *via* environmentally friendly policies. In further comprehending this notion, research conducted by Ain and Yousaf also presents a sustainable view of facilitating individuals' mental health and wellbeing. The study states that new approaches to mental health fortify individuals' strength in managing the emerging psychological challenges. It suggests that fiscal decentralization impacts the individuals' psychological wellbeing, thus improving the effectiveness of the employees' service.

However, several psychological theories have added to the need for positive employees' mental health. In recent years, the global health emergency has boosted the demand for proactive health measures, thus combatting the increasing health crises. Despite threatening the lives of the Chinese population, the virulence of coronavirus has made anti-epidemic approaches to control the devastating effect of the pandemic (i.e., medical instruments and surgical materials) (Wilder-Smith and Freedman, 2020). In illustration, Tang et al. state that anti-pandemic materials have curbed the epidemic crises by ensuring the optimal allocation of resources to the general public.

With the progressing development of this domain, technological advancement has fundamentally proposed extensive implications for individuals' health. Today, the new wave of technology has supported the psychological phenomenon by leading the digital transformations to be an integral mechanism for achieving sustainable development. In the modernization age, the study conducted by Li indicates digitalization to be a vital component in establishing team innovation and digital culture, thus ensuring the organization's sustainable performance. Moreover, in today's business world, moral ethics also plays a vital role in promoting the organizations' sustainability and welfare. Accordingly, another study conducted by Guo states that the evolution of modern leadership in the digital business world has shaped the organizational working processes, thereby bringing massive health advantages.

Unsurprisingly, today information technology has profoundly improved the employees' psychological wellbeing. Previously, the literature highlighted the dark side of using off-time work-related technology on employees' health (i.e., poor sleep quality) (Gadeyne et al., 2018). In compassion, the technological research featured by Hu et al. found that off-time work-related technology (e.g., smartphone) positively influences bedtime procrastination, thus increasing self-control depletion. Undoubtedly, too much interdependence on the technology may lead to poor employees' mental health. Accordingly, the study suggested by Hang et al. supports the use of technology inhibitors, helping employees reduce the increasing effect of workplace technostress. In line with the positive occupational health psychology, the study by Zhang reveals that teachers play a vital role in nurturing students' psychological growth. In recent years, psychological wellbeing has become the core concern of teachers. Hence, this study shows that teacher self-abilities regulate the students' self-learning skills, wellness, and academic performance.

Altogether, these papers indicate occupational health psychology as the fastest-growing discipline capturing the researchers' attention. Its rapid growth during the last decades has built a new wave of pioneer research, inspiring future researchers to establish new grounds for innovations. This call for the paper had unpacked the challenges and opportunities by enhancing the future perception regarding the OHP. This interdisciplinary approach has enabled multi- theoretical research to publish in the journal frontier of psychology. We hope that all these papers have facilitated the growth in the discipline while providing guidelines to scientists, practitioners, organizations, professionals, and policymakers. Indeed, we expect these articles to inspire future researchers and scholars to explore novel opportunities in the OHP domain.

## Author contributions

All authors listed have made a substantial, direct, and intellectual contribution to the work and approved it for publication.

## Conflict of interest

The authors declare that the research was conducted in the absence of any commercial or financial relationships that could be construed as a potential conflict of interest.

## Publisher's note

All claims expressed in this article are solely those of the authors and do not necessarily represent those of their affiliated organizations, or those of the publisher, the editors and the reviewers. Any product that may be evaluated in this article, or claim that may be made by its manufacturer, is not guaranteed or endorsed by the publisher.
